# Supplementary material for: Review of the Current Status on Ruminant Abortigenic Pathogen Surveillance in Africa and Asia
Source: Vet Sci. 2024 Sep 12;11(9):425. doi: 10.3390/vetsci11090425 (PMC11435614; doi:10.3390/vetsci11090425)
Supplement: Supplementary file 1 [file vetsci-11-00425-s001.zip › vetsci-2924002-supplementary.pdf]

Supplementary Materials

Table S1. Criteria list fulltext.

Criteria	Key element	Comments
Cattle, Goats, Sheep	Population	Studies on the species of interest only.
Abortifacient organisms	Intervention/Exposure	Organisms of interest as outlined above.
Abortion	Outcome	Abortion in livestock spp of interest.
Africa and Asia	Region	Only in regions of interest
Surveillance technologies	Surveillance type	
Only English	Language	
Sample used for surveillance	Sample type during surveillance	
Laboratory methodology used to confirm cases	Lab methodology	Any lab confirmation of cases done.
Linkage to formal surveillance system in the region	Survey	Either linked to government systems or independent studies.
Regions	Comparator	Systems will be compared regionwise.

Table S2. Criteria list title/abstract.

Criteria	Key element	Comments
Cattle, Sheep, Goats	Population	Any paper that mentions ALL or ANY of these species will be included.
Abortifacient organisms commonly found in Africa and Asia.	Intervention/Exposure	Any
Abortion surveillance	Outcome	Surveillance or studies in support of surveillance of any of the mentioned common abortifacient agents.
English Language	Language	All articles/ abstracts should be in English language.
Africa and Asia	Geographical Region	
Primary Information	Primary peer-reviewed article	Reviews and any article without primary information will be excluded.
None	Comparator	None
